# *De Novo* RNA Sequencing and Transcriptome Analysis of *Monascus purpureus* and Analysis of Key Genes Involved in Monacolin K Biosynthesis

**DOI:** 10.1371/journal.pone.0170149

**Published:** 2017-01-23

**Authors:** Chan Zhang, Jian Liang, Le Yang, Baoguo Sun, Chengtao Wang

**Affiliations:** 1 School of Food and Chemical Engineering, Beijing Technology & Business University, Beijing, China; 2 Beijing Engineering and Technology Research Center of Food Additives, Beijing Technology & Business University, Beijing, China; 3 Beijing Key Laboratory of Flavor Chemistry, Beijing Technology &Business University, Beijing, China; Huazhong University of Science and Technology, CHINA

## Abstract

*Monascus purpureus* is an important medicinal and edible microbial resource. To facilitate biological, biochemical, and molecular research on medicinal components of *M*. *purpureus*, we investigated the *M*. *purpureus* transcriptome by RNA sequencing (RNA-seq). An RNA-seq library was created using RNA extracted from a mixed sample of *M*. *purpureus* expressing different levels of monacolin K output. In total 29,713 unigenes were assembled from more than 60 million high-quality short reads. A BLAST search revealed hits for 21,331 unigenes in at least one of the protein or nucleotide databases used in this study. The 22,365 unigenes were categorized into 48 functional groups based on Gene Ontology classification. Owing to the economic and medicinal importance of *M*. *purpureus*, most studies on this organism have focused on the pharmacological activity of chemical components and the molecular function of genes involved in their biogenesis. In this study, we performed quantitative real-time PCR to detect the expression of genes related to monacolin K (*mokA-mokI*) at different phases (2, 5, 8, and 12 days) of *M*. *purpureus* M1 and M1-36. Our study found that *mokF* modulates monacolin K biogenesis in *M*. *purpureus*. Nine genes were suggested to be associated with the monacolin K biosynthesis. Studies on these genes could provide useful information on secondary metabolic processes in *M*. *purpureus*. These results indicate a detailed resource through genetic engineering of monacolin K biosynthesis in *M*. *purpureus* and related species.

## Introduction

*Monascus purpureus* is a mold that is an important medicinal and edible resource. Products fermented with *M*. *purpureus* have been used for thousands of years in China and other Southeast Asian countries. *M*. *purpureus* metabolic components include bioactive compounds [1–3] such as pigments [4], monacolin K [5–7], citrinin [8], aminobutyric acid [9], ergosterol, toxins and other metabolites [10,11], hydrolytic enzymes [12] that are involved in a wide range of activities [13,14] and are used as ingredients in food coloring [15,16], wine and pharmaceuticals.

Red yeast rice (RYR) is produced by *M*. *purpureus* fermentation and is also known as red koji [17,18], Hung-Chu, Hong Qu, Ang-kak, Ankak rice, Red Mold rice, or Beni-Koji. RYR has long been recognized for its effects in lowering cholesterol [19,20] and improving resistance of bacteria [21]. In East Asian countries, RYR is traditionally used as a food flavoring agent, coloring agent, preservative, and as a health product to maintain lipid profiles and blood pressure, for instance by normalizing c-reactive protein levels in hypertension [22]. *M*. *purpureus* is a commercially important organism owing to its production of the medicinal ingredient monacolin K [23]; the biosynthesis pathway for this compound along with those of citrinin [24] and pigments (fungal polyketone compounds) has been linked to polyketide synthase.

Currently, only about 889 nucleotide sequences and 37 expressed sequence tags (ESTs) can be found for *M*. *purpureus* in GenBank (National Center for Biotechnology Information). Moreover, there is little information on functional genes in *M*. *purpureus*. The transformation efficiency of *M*. *purpureus* has recently improved, which is expected to aid in the generation of genetically modified *M*. *purpureus* cell lines. However, the lack of genomic sequence information especially information regarding those of functional genes remains a major obstacle.

High-throughput sequencing has revolutionized genomics research with its low cost and ultra-high data output, including the parallel production of millions of short cDNA reads. RNA sequencing (RNA-seq) is a high-throughput approach that can be used to determine transcript abundance [25] and identify novel transcriptionally active regions [26] even in single cells. This technique is especially important for analyzing the transcriptomes of non-model species [27,28] because it does not require prior knowledge of transcript sequences.

In this study, we investigated the transcriptome of *M*. *purpureus* by RNA-seq [29–31], which was then annotated using publicly available databases and tools to identify genes involved in the biosynthesis of fungal polyketone compounds that may be related to the tricarboxylic acid cycle [32]. To this end, mRNA was isolated from liquid medium during vegetative growth of *M*.*purpureus*, and was used to generate an RNA-seq library. Unigenes were then assembled de novo from sequenced short reads and annotated using the Basic Local Alignment Search Tool for proteins (BLASTX). Gene Ontology (GO) classification and pathway analysis based on the Kyoto Encyclopedia of Genes and Genomes (KEGG) database was also performed[33–38]. The results in our study provide a foundation for the functional characterization of *M*. *purpureus* genes.

We performed quantitative real-time PCR (qRT-PCR) to detect the expression of genes related to monacolin K (*mokA*-*mokI*) at different phases (2, 5, 8, and 12 days) of *M*. *purpureus* M1 and M1-36 to find the key genes related to monacolin K biogenesis in *M*. *purpureus*. The qRT-PCR technology provides a powerful and efficient method for gene function analysis in *M*. *Purpureus*.

## Materials and Methods

### *M*. *purpureus* culture preparation

*M*. *purpureus* was obtained from The Chinese General Microbiological Culture Collection Center (Strain Number, CGMCC 3.0568), China. *M*. *purpureus* M1 was a wild-type strain. *M*. *purpureus* M1-36 was a high producer of monacolin K strain, based on the M1 by mutagenesis. M1 and M1-36 were reactivated by streaking on potato dextrose agar plates from a frozen glycerol stock and were grown for 2–3 days.

Liquid cultures were grown for 2–3 days in a 250 ml flask at 30°C with shaking at 220 rpm. For liquor fermentation, cells were cultured in a 250 ml flask at 30°C with shaking at 150 rpm for 2 days for followed by 25°C with shaking at 150 rpm for 10 days.

### *M*. *purpureus* collection and RNA isolation

High monacolin K-producing *M*. *purpureus* (M1-36) and wild-type *M*. *purpureus* (M1) were collected at two growth phases namely rowing and maturation during which secondary metabolites are produced. Cells were immediately frozen in liquid nitrogen and stored at -80°C until use. Total RNA was isolated using the RNAprep Pure Plant kit (Polysaccharide & Polyphenolic-rich) and DNase I was used to remove contaminating DNA. RNA quality was analyzed using a 2100 Bioanalyzer (Agilent Technologies, Santa Clara, CA, USA). Oligo (d) T beads (Qiagen, Hilden, Germany) were used to isolate poly(A) mRNA from total RNA.

### RNA-seq library preparation and sequencing

To construct the RNA-seq library, 2.5 μg of RNA from four biological replicates was pooled. mRNA enrichment, fragment interruption, addition of adapters, size selection, PCR amplification, and RNA-Seq were performed by LifeTech Co. (Beijing, China). Poly(A) mRNA was isolated using oligo d(T) beads and broken into short fragments. A paired-end RNA-seq library was prepared and sequenced on the Illumina HiSe 2000 platform according to the manufacturer’s instructions. In total, 5 G of raw data was obtained.

### Analysis of illumina sequencing results

“Dirty” raw reads (i.e., those with adaptors of low quality, with > 5% unknown nucleotides) were discarded. De novo assembly of short reads (from the paired-end RNA-seq library) into a transcriptome was performed with previously described software using Trinity as the default parameter [39]. We first removed low-quality reads from the raw data set and used the Trinity assembler to obtain transcript references; we then aligned the clean reads to the de novo assembly reference. The EdgeR module was used to evaluate differences in expression. Transcripts were aligned with BLASTX, and annotations were performed using the Swiss-Prot database. Only transcripts with E < 1e^-5^ were selected.

### Annotation and classification of unigenes

All unigenes were annotated by a BLASTX search against NCBI nonredundant protein(Nr), Swiss-Prot, Translated European Molecular Biology Laboratory Nucleotide Sequence, COG, NCBI nucleotide database (Nt) and KEGG databases by using a cut-off E-value of 1025. Based on the results of the database annotation, we selected the protein database with the highest sequence similarity to the function annotations. Unigenes were assigned to different pathways by searching against the KEGG databases. Based on the results of the Nr database annotation, the Blast2 GO program was used to obtain the GO annotation of assembled unigenes. WEGO software was then used to plot the GO functional classification for unigenes with a GO term hit to visualize the distribution of gene functions at the macro level.

### Confirmation of unigenes assembled based on the RNAseq data by cDNA and genomic DNA amplification and sequencing

Total RNA was isolated from thalli during the rowing phase, using RNAprep Pure Plant kit (Polysaccharide & Polyphenolic-rich) and removed DNA. Total RNA (500 μg-2 mg) was reverse- transcribed into cDNA using the FastQuant RT Kit (Tiangen, Beijing, China) following the manufacturer’s instructions. cDNA fragments were amplified on a Mastercycler (Eppendorf, Hamburg, Germany) using unigene-specific primers, that were designed using Primer Premier 5 software (Premier Biosoft, Palo Alto, CA, USA). The PCR reaction (20 μl) consisted of cDNA template (2 μl), 10× buffer (2 μl), primers (2 mM, 2 μl), MgCl_2_ (2.5 mM, 2 μl), Taq DNA polymerase (5000 U/μl, 0.2 μl), dNTPs (2 mM, 2 μl), and ddH_2_O (9.8 μl). PCR products were separated on a 1% agarose gel and bands were excised and gel-purified before being sequenced by Invitrogen (Shanghai, China). Genomic DNA fragments corresponding to cDNAs were amplified using DNA extracted by the cetyltrimethylammonium bromide method and purified and sequenced. cDNAs and corresponding genomic DNAs were aligned to identify introns in the unigenes.

### Identification of transcription factors and target genes in *M*. *purpureus*

To identify the transcription factors, all unigenes were searched using Transdecoder analysis tool (http://www.transcriptionfactor.org). The high-throughput sequencing results were predicted by Open Reading Frame (ORF) to obtain the protein, using the Transdecoder software [40]. The HMM motif of the transcription factor was downloaded and predicted by hmmscan in HMMER software to obtain the transcription factor information. Only transcripts with E < 1e^-5^ were selected.

To extract the sequence from the upstream of transcription initiation site (100–5000 bp) as the promoter region for further search. The site weight matrix motif of the transcription factor were downloaded from http://cisbp.ccbr.utoronto.ca, and the upstream sequences were predicted by the mast program in the MEME software to obtain the target genes [41,42].

### Monacolin K detection by HPLC

Moncolin K was detected by HPLC under the following conditions: C18 chromatographic column (5 microns, 150 mm × 4.6 mm); acetonitrile:0.1% phosphoric acid (v/v) = 65:63 as the mobile phase; column temperature 25°C; flow rate 1 ml/min; wavelength = 238 nm.

### qRT-PCR analysis of key genes in monacolin K biosynthesis

*M*. *purpureus* M1-36 and M1 gene expression profiles were determined by qRT-PCR on a CFX96 qRT-PCR detection system (Bio-Rad, Hercules, CA, USA) with associated software.

cDNA reverse-transcribed from total RNA was used as the template along with SuperReal PreMix Plus with SYBR Green (Tiangen Biotech, Beijing, China) and unigene-specific primers (Table 1) designed with Beacon Designer 8 software (Premier Biosoft). The amplification program was as follows: 95°C for 15 min, followed by 40 cycles of 95°C for 10 s, 60°C for 30 s, and 72°C for 30 s. Relative abundance of the transcripts was calculated by the comparative cycle threshold method with GAPDH used as an endogenous control. Reactions were prepared in triplicate for each sample.

**Table 1:** Target genes and primers for *M*. *purpureus*.

Genes	Primer pairs	length(bp)	Tmvalue
*mokA*	5’- GACCTCGGTCATCTTGGC -3’	18	59.1
	5’- TTGTTCCAAGCGGTCTTC -3’	18	57.3
*mokB*	5’- AAACATCGTCACCAGTCT -3’	18	50.7
	5’- CTAAGTCGGGCATCTACC -3’	18	53.1
*mokC*	5’- CAAGCTGCGAAATACACCAAGCCTC -3’	25	63.6
	5’- AGCCGTGTGCCATTCCTTGTTGTCC -3’	25	65.3
*mokD*	5’-TTCATCTGCTGCTGGTAT -3’	18	59.8
	5’-AACTTCTCACCGTCAATG -3’	18	58.7
*mokE*	5’- ATCGCAGGTCACGCACATCCAAGTC -3’	25	72.3
	5’- GTAAAGGCAGCCCGAGCAGCTTCAT -3’	25	71.1
*mokF*	5’- GAGATCATAGTGGCCGACTGAA -3’	22	59.8
	5’- ACCGTCTCATCCAACCTCACGA -3’	22	56.1
*mokG*	5’- CCAGGTAACCAACGGATTA -3’	19	56
	5’- GATCAGAGCAGTCACCAG -3’	18	52
*mokH*	5’- CAGGAAATCTGGACTTACCCCATTG -3’	25	65.8
	5’- TGTTGGATTGTTGTTGGAGATATAC -3’	25	59.2
*mokI*	5’-ATGTTGAATGGCAATGATGG -3’	20	60.9
	5’-CAGCGTGGGTGATGTATC -3’	18	61.7
*GAPDH*	5’- CCGTATTGTCTTCCGTAAC -3’	19	55.4
	5’- GTGGGTGCTGTCATACTTG -3’	19	57.6

## Results and Discussion

### *De novo* transcriptome assembly and generation of unigenes

**Fig 1:**
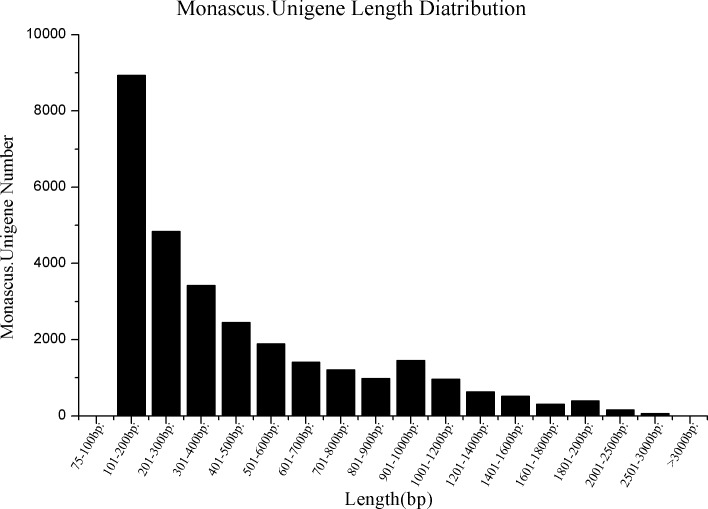
Length distribution of the unigene library of *M*. *purpureus*.

Samples were collected from rowing and maturation phases. Five cDNA libraries were constructed (M0, M1-1, M1-2, M3, and MW) from approximately 10.6, 12.8, 20.6, 12.7, and 17.9 million raw reads of 200 bp, respectively. In total, 74,557,640 original reads were generated using the HiSeq2000 sequencing platform (Illumina, San Diego, CA, USA). The sequencing error rate (Q20 = 1%) was > 94.00% and the average GC content was 50.33%. Adaptor and duplicate sequences and ambiguous and low-quality reads were removed, and high-quality reads for each cultivar were separately assembled using the NGS QC Toolkit v.2.3 [43] and a series of stringent criteria. After this quality control step, 60,348,338 high-quality reads with a length of 101 bp (average length: 86.62bp) were retained (Fig 1): these were used to assemble contigs and obtain transcripts in default parameter settings by the Trinity program [44]. In total, 41,002 contigs were generated with a k-mer of 25, which was pre-defined to avoid misassembly caused by a too-short k-mer while retaining a reasonable number of reads [45,46]. In the step of assembling, 41,002 transcripts had a median size (N50) of 900 bp and 29,713 unigenes had an N50 of 789 bp from all of the contigs; 39% of the unigenes were not shorter than 500 bp and approximately 200 (0.80%) were longer than 2 kb. In our study, the total length of the assembled transcriptome was 17.8 Mbp (Table 2).

**Table 2:** *De novo* assembly of the *M*. *purpureus* transcriptome.

Feature	Number of features					Total length	N50 (bp)	Mean length(bp)
	<0.5 kb	0.5–1 kb	1–2 kb	>2 kb.	Total			
Contig	25,024	10,446	5,057	475	41,002	27,191,301	900	663
	(61.03%)	(25.48%)	(12.33%)	(1.16%)				
Unigene	19,674	6,968	2,833	238	29,713	17,834,993	789	600
	(66.21%)	(23.45%)	(9.53%)	(0.80%)				

### Annotation and classification of *M*. *purpureus* unigenes

To identify the putative functions of the assembled unigenes, we determined all of the unigenes by a BLAST search of various databases [47,48]. The unigenes were first searched against the National Center for Biotechnology Information (NCBI; Bethesda, MD, USA) non-redundant protein database (Nr) using BLASTX with a cut-off of P<0.05 and 丨log2丨≥1. The possibility of misassembly was negligible because of the relatively long k-mer (k = 25). In the end, a high percentage of unigenes, i.e., 21,331 of 29,713(71.8%), had a match in this database. A portion of the 8,382 (28.2%) unigenes that did not have a match were presumed to be unique to *M*. *purpureus*. Of the 21,331 unigenes with an orthologous match, 15,394 (72.2%) had a match with an annotated function, whereas the remainder had a match that was classified only as a predicted protein.

**Fig 2:**
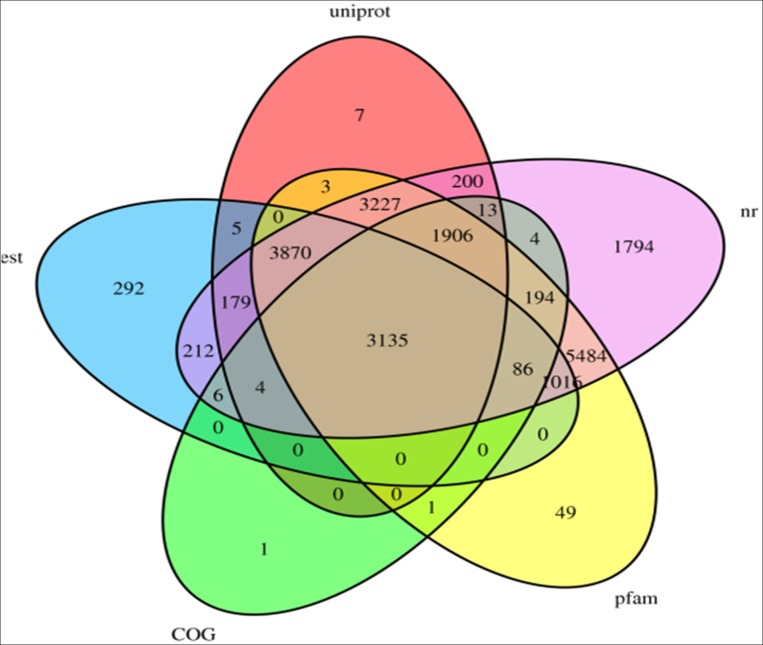
Transcript homology of five *M*. *purpureus* cultivars and Venn diagram of the number of orthologous transcripts (E-value = 1e^-5^). Venn diagram includes 31 possible intersections between five libraries, and the number of genes shows in each of these overlapping categories. Transcriptome data sets are as follows: COG (light-green color), pfam (light-yellow color), Nr (light-purple color), Uniprot (light-pink color), and Est (light-blue color), the immature embryos.

Additional BLAST searches revealed 5,351, 8,806, 21,331, 18,972, and 12,550 unigenes with matches in the Clusters of Orthologous Groups (COG), Expressed Sequence Tags (EST), Nr, Protein Family (Pfam), and Uniprot databases, respectively (Table 3). In total, 5,351 unigenes had a least match term with the COG databases, whereas 21,331 had a hit against the Nr database (Fig 2).

**Table 3:** Summary of annotations for the *M*. *purpureus* unigene library.

Anno database	Annotated number
COG Annotation	5351
Est Annotation	8806
Nr Annotation	21331
Pfam Annotation	18972
Uniprot Annotation	12550

**Fig 3:**
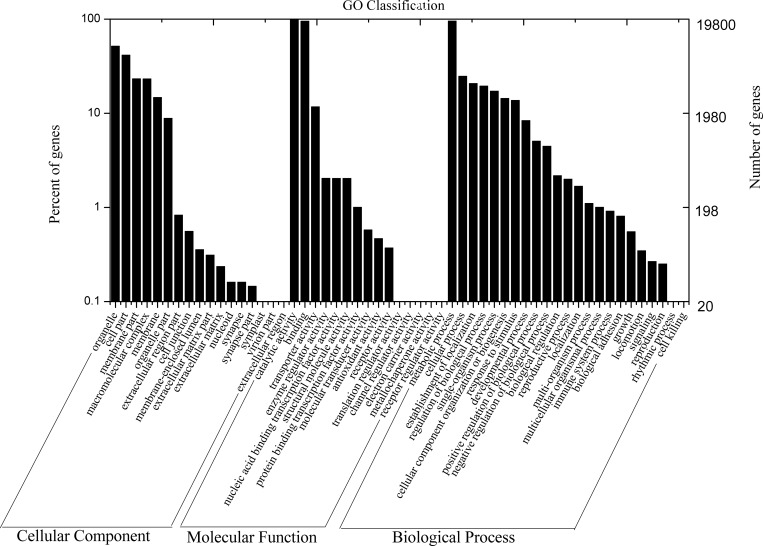
GO classification of the *M*. *purpureus* unigene library.

In a further analysis of gene function, 122,599 unigenes showed a match with the GO database [49]. These unigenes were categorized into 55 functional subgroups belongs to three main GO groups, i.e., cellular component, molecular function, and biological process (Fig 3). We found that the most frequent GO terms in the groups were organelle (10,300 unigenes), catalytic activity (19,800 unigenes), and metabolic process (19,007 unigenes).

Of the 29,713 *M*. *purpureus* unigenes, 19,242 were classified into 26 clusters based on COG analysis. Based on these results, we found that majority of these unigenes belonged to “general function prediction” cluster (n = 2,478; 12.88%), followed by “translation, ribosomal structure, and biogenesis” (n = 2,122; 11.03%); “energy production and conversion” (n = 1,992; 10.35%); “amino acid transport and metabolism” (n = 1,920; 9.98%);“carbohydrate transport and metabolism” (n = 1828; 9.50%); “posttranslational modification, protein turnover, chaperones” (n = 1,652; 8.59%); and “lipid transport and metabolism” (n = 1,348; 7.01%). Clusters represented by the fewest unigenes were “intracellular trafficking, secretion, and vesicular transport” (n = 28; 0.15%); “chromatin structure and dynamics” (n = 22; 0.14%); “cell motility” (n = 12; 0.06%); and “RNA processing and modification” (n = 2;0.01%). None were related to “nuclear structure”, “cytoskeleton” or “extracellular structures” (Fig 3).

**Fig 4:**
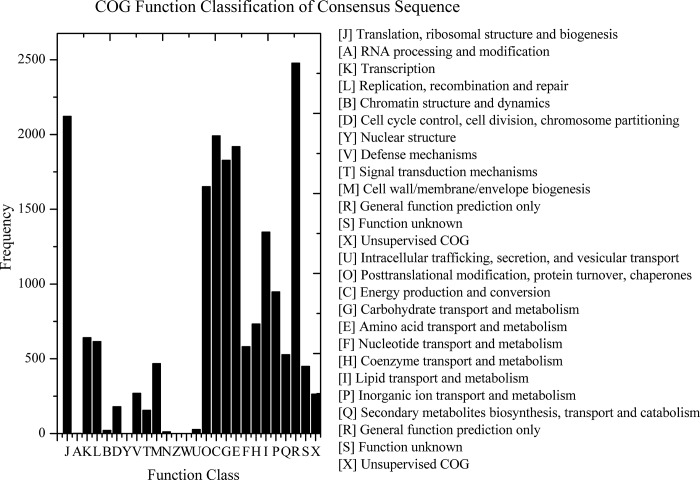
COG functional classification of *M*. *purpureus* unigenes based on KEGG pathway analysis.

To examine gene interactions and biological functions in *M*. *purpureus*, the unigenes were searched against reference canonical pathways in KEGG. In total, 13,565 unigenes were assigned to 306 pathways. The most highly represented of these were “metabolic pathways” (ko01100, n = 1589, 11.71%), “biosynthesis of secondary metabolites” (ko01110, n = 624, 4.60%), “microbial metabolism in diverse environments” (ko01120, n = 421, 3.10%), “spliceosome” (ko03040, n = 202, 1.49%), “purine metabolism” (ko00230, n = 201, 1.48%), “RNA transport” (ko03013, n = 199, 1.47%), “protein processing in endoplasmic reticulum” (ko04141, n = 195, 1.44%), “ribosome” (ko03010, n = 183, 1.35%), “cell cycle-yeast” (ko04111, n = 178, 1.31%), “oxidative phosphorylation” (ko00190, n = 174, 1.28%), “ribosome biogenesis in eukaryotes” (ko03008, n = 153, 1.13%), “HTLV-I infection” (ko05166, n = 151, 111%), “pyrimidine metabolism” (ko00240, n = 150, 1.11%), “Huntington's disease” (ko05016, n = 150, 1.11%), “Epstein-Barr virus infection”(ko05169, n = 142, 1.05%), “ubiquitin mediated proteolysis” (ko04120, n = 141, 1.04%),“cell cycle” (ko04110, n = 130, 0.99%), “Parkinson's disease” (ko05012, n = 129, 0.95%), “meiosis—yeast” (ko04113, n = 127, 0.94%), “glycolysis/gluconeogenesis” (ko00010, n = 120, 0.88%), and “Alzheimer's disease” (ko05010, n = 120, 0.88%) (Fig 4).

**Fig 5:**
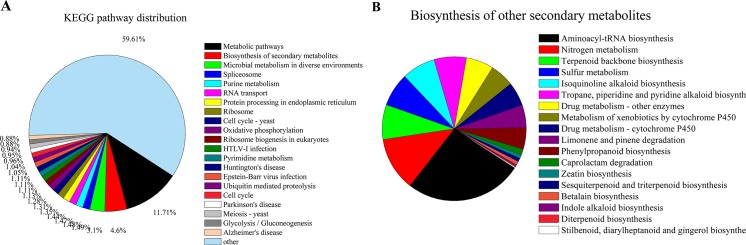
Pathway assignment based on KEGG mapping.

The “biosynthesis of other secondary metabolites” pathway included unigenes for “aminoacyl-tRNA biosynthesis” (n = 98); “nitrogen metabolism” (n = 45); “terpenoid backbone biosynthesis” (n = 29); “sulfur metabolism” (n = 28); “isoquinoline alkaloid biosynthesis” (n = 28); “tropane, piperidine and pyridine alkaloid biosynthesis” (n = 27); “drug metabolism-other enzymes” (n = 23); “metabolism of xenobiotics by cytochrome P450” (n = 20); “drug metabolism-cytochrome P450” (n = 20); “limonene and pinene degradation” (n = 19); “phenylpropanoid biosynthesis” (n = 18); “caprolactam degradation” (n = 5); “zeatin biosynthesis” (n = 3); “sesquiterpenoid and triterpenoid biosynthesis” (n = 3); “betalain biosynthesis” (n = 3); “indole alkaloid biosynthesis” (n = 1); “diterpenoid biosynthesis” (n = 1); and “stilbenoid, diarylheptanoid and gingerol biosynthesis” (n = 1) (Fig 5).

### Differential gene expression in the *M*. *purpureus* libraries

**Fig 6:**
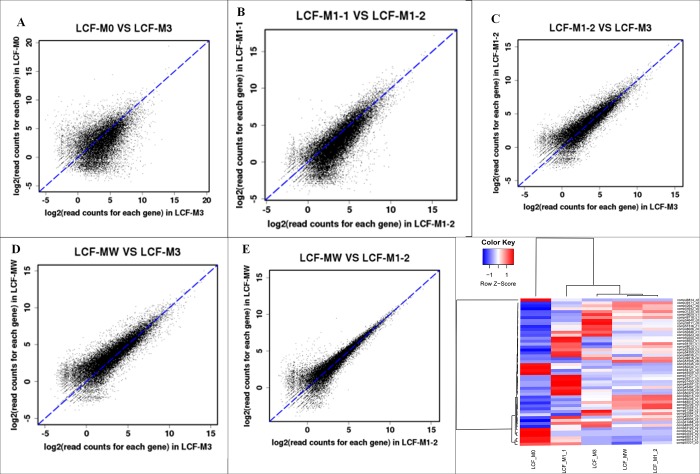
**Distribution of genes differentially expressed between two M.purpureus cultivars (A-E) and heatmap analysis of top 50 highly expressed unigenes in both samples (F).** (A) M0 vs. M3. (B)M1-1 vs. M1-2. (C)M1-2 vs. M3. (D)MW vs. M3. (E)MW vs. M1-2. (F) Clustering of top 50 genes expression profiles at 5 different comparisons. Every colum represent the DEGs in different samples. Red color represents increasing level of the gene expression and blue color indicates decreasing of the gene expression. Each row represents a gene.

We measured the transcript abundance in each sample and identified genes that were differentially expressed between the two *M*. *purpureus* cultivars. Clean reads from each sample were mapped to the constructed reference genes, and mapped reads were counted to obtain reads per kilobase per million mapped reads values. In total, 273 differentially expressed genes were detected in the five *M*.*purpureus* libraries, of which 12482, 12487, 12428, 12520, and 12520 were predicted from “LCF-M0 vs. LCF-M3”, “LCF-M1-1 vs. LCF-M1-2”, “LCF-M1-2 vs. LCF-M3”, “LCF-MW vs. LCF-M3”, and “LCF-MW vs. LCF-M1-2”, respectively (Fig 6A–6E).

Heap map of top 50 differentially expressed genes in the five samples prepared using the MeV (MultiExperiment Viewer) program (Fig 6F). The left panel of Fig 6F shows the calculated log2 for clustering of the top 50 differentially transcribed genes at different stages of M1 and M1-36. It shows significant transcriptional differences. Compared with M1-2, the function of up-regulation genes in M3 were mainly concentrated in aspects: metabolic pathways, microbial metabolism in diverse environments, aminoacyl-tRNA biosynthesis, purine metabolism, alanine, aspartate and glutamate metabolism, cysteine and methionine metabolism. Compared with M1-2, the function of down-regulation genes in M3 were mainly concentrated in aspects: metabolic pathways, biosynthesis of secondary metabolites, starch and sucrose metabolism, pyruvate metabolism, fatty acid biosynthesis, methane metabolism.

### Identification of transcription factors and target genes in *M*. *purpureus*

**Fig 7:**
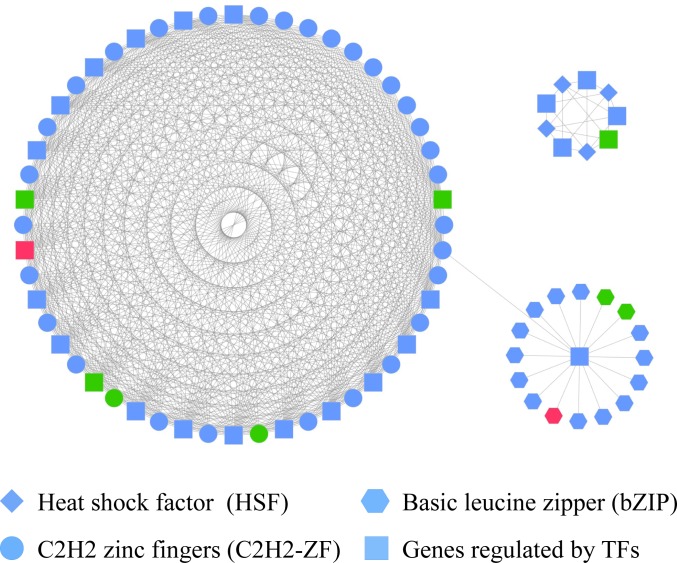
Construction of TF–based regulation network in the *M*. *purpureus*. Round, rhombus, hexagon represented three different transcription factors, and squares represented the regulated gene. Green represented the down-regulated genes, and red represented the up-regulated genes.

Transcription factors (TFs) can affect the expression of many genes, also can guide a lot of different biological processes, including cell differentiation, secondary metabolism and so on. Therefore, the identification of transcription factors and target genes will help us obtain a better understanding of genes regulatory networks on monacolin K biosynthesis in *M*. *purpureus*. 15,037 protein sequences were obtained after ORF prediction and the default filter screening. 125 transcription factors were finally selected with E < 1e^-5^ filtering. Finally, 52 transcription factors and 27 target genes were obtained, which were screened according to the p <1e^-8^(Fig 7). In Fig 7, the genes in M3 sample were used as the experimental group, and the genes in M1-2 sample as the control group. It could be seen that 4 transcription factors were down-regulated and 1 transcription factors was up-regulated in M3 strain (Table 4). There were also 4 down-regulated target genes and 1 up-regulated target gene in M3 strain (Table 4).

**Table 4:** Down & up regulated genes of transcription factors and target genes in *M*. *purpureus* unigene library.

Gene_ID	Type	uniprot_description	Gene length	RNA name	DEGs in M3vsM1-2
comp51725_c0	bZIP	—	—	—	down
comp53355_c1	bZIP	P52890	3669	Transcription factor atf1	up
comp53366_c10	bZIP	P78962	2220	Transcription factor atf21	down
comp54181_c0	C2H2-ZF	Q9Y815	4529	Zinc finger protein rsv2	down
comp54338_c3	C2H2-ZF	Q9BXK1	252	Krueppel-like factor 16	down
comp48940_c0	Regulated by TFs	P49373	1523	Transcription elongation factor S-II	down
comp50904_c4	Regulated by TFs	—	—	—	down
comp52324_c4	Regulated by TFs	—	—	—	down
comp52338_c2	Regulated by TFs	Q99002	262	14-3-3 protein homolog	up
comp53402_c0	Regulated by TFs	Q9HGY9	362	Fructose-bisphosphate aldolase	down

### Monacolin K contents in five *M*. *purpureus* cultivars determined by high-performance liquid chromatography (HPLC)

**Fig 8:**
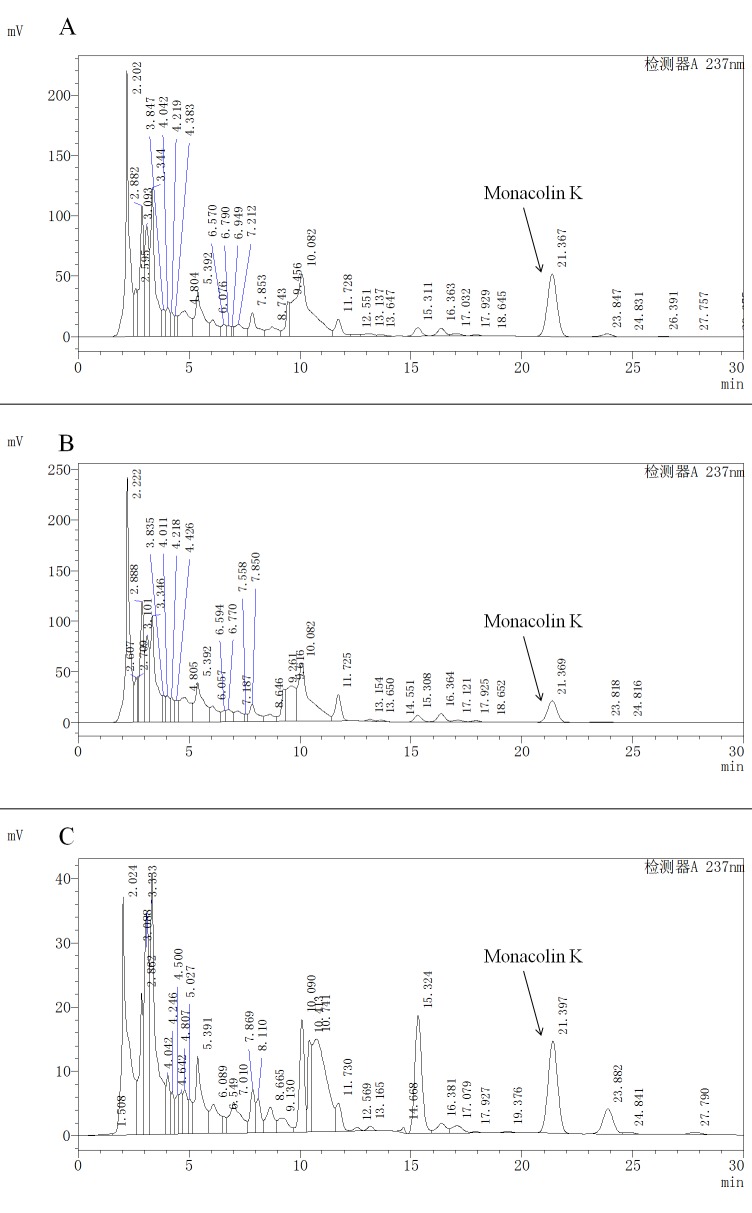
The content of monacolin K were detected by HPLC in different *M*.*purpureus* cultivars. (A) M3 was from the stabilization phases of M1-36. (B) M1-2 was from the stabilization phases of M1. (C) MW was the maximum monacolin K biosynthesis quality phase of M1. In the HPLC detection system, monacolin K peak appears about 21.300 min in every 30 minutes.

We determined from the growth curve of *M*. *purpureus* that the adjustment, logarithmic, and stabilization phases were 2, 5, and 12 days, respectively. Monacolin K biosynthesis [50,51] was maximal at 8 days. Monacolin K was extracted from five fractions of *M*. *purpureus*–fermented liquor and determined by HPLC. The monacolin K content and proportions differed in the five *M*. *purpureus* samples, and five samples represented different stages of M1 and M1-36. M1-1 and M1-2 were produced in M1 adjustment and stabilization phases, respectively, whereas M0 and M3 were from the adjustment and stabilization phases of M1-36, and MW was the maximum monacolin K biosynthesis quality phase of M1. No monacolin K was detected in M0 and M1-1, and the content was higher in M3 (295.6101 mg/l) than in M1-2 (117.7263 mg/l) and MW (83.9650 mg/l) among the five *M*. *purpureus* cultivars. Monacolin K contents were higher in M1-2 and M3 than in M1-1, M0, and MW (Fig 8).

### Genes expression patterns and biosynthesis pathway analysis of monacolin K in *M*. *purpureus*

**Fig 9:**
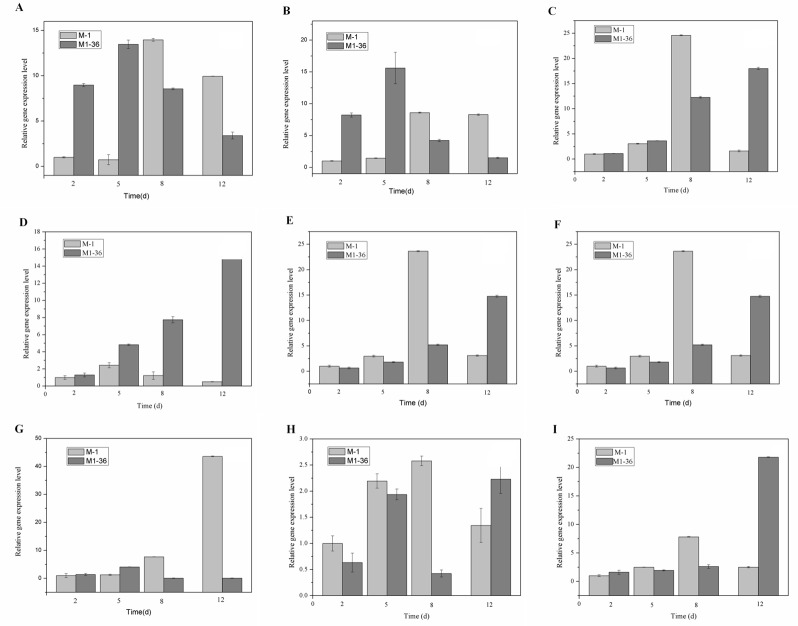
Expression of genes related to monacolin K (*mokA-mokI*) at different phases (2, 5, 8, and 12 days) of *M*. *purpureus* M1 and M1-36. Bar graphs illustrate gene expression levels at different times and in different strains. (A) *mokA*; (B) *mokB*; (C) *mokC*; (D) *mokD*; (E) *mokE*; (F) *mokF*; (G) *mokG*; (H) *mokH*; and (I) *mokI*. Data represent the mean ± SD of three independent biological replicates.

In this study, nine important genes involved in the biosynthetic pathway of monacolin K, i.e., *mokA* (polyketide synthase), *mokB* (polyketide synthase), *mokC* (P450 monooxygenase), *mokD* (oxidoreductase), *mokE* (dehydrogenase), *mokF* (transesterase), *mokG* (HMG-CoA reductase), *mokH* (transcription factor) and *mokI* (efflux pump). The expression of genes related to monacolin K at different phases of *M*.*purpureus* growth was analyzed by quantitative real-time PCR (qRT-PCR) [52]. Expression levels gradually increased at 2, 5, and 8 days, but decreased at 12 days (Fig 9).

For example, in M1, *mokC* transcript levels were 3.05-, 24.59-, and 1.60-fold higher; *mokD* transcript levels were 2.43-, 1.23-, and 0.50-fold higher; *mokF* transcript levels were 2.97-, 23.64-, and 3.10-fold higher; and *mokI* transcript levels were 2.49-, 7.82-, and 2.49-fold higher than those in the controls at 5, 8, and 12 days, respectively (Table 5). For M1-36, *mokC* transcript levels were 1.11-, 3.62-, 12.27-, and 18.00-fold higher; *mokD* transcript levels were 1.30-, 4.82-, 7.75-, and 15.71-fold higher; *mokF* transcript levels were 0.65-, 1.81-, 5.22-, and 14.76-fold higher; and *mokI* transcript levels were 1.62-, 1.94-, 2.61-, and 21.81-fold higher than in the control at 2, 5, 8, and 12 days, respectively (Table 5).

**Table 5:** Expression of genes related to monacolin K (*mokA-mokI*) at different phase (2, 5, 8, and 12 days) of *M*. *purpureus* M1 and M1-36*.

genes	M1				M1-36			
	2d	5d	8d	12d	2d	5d	8d	12d
*mokA*	1.00	0.73	13.96	9.94	8.98	13.46	8.55	3.40
*mokB*	1.00	1.44	8.59	8.28	8.22	15.60	4.23	1.51
*mokC*	1.00	3.05	24.59	1.60	1.11	3.62	12.27	18.00
*mokD*	1.00	2.43	1.23	0.50	1.30	4.82	7.75	15.71
*mokE*	1.00	12.64	43.11	29.18	0.01	0.24	0.16	0.22
*mokF*	1.00	2.97	23.64	3.10	0.65	1.81	5.22	14.76
*mokG*	1.00	1.27	7.67	43.61	1.39	4.05	0.05	0.04
*mokH*	1.00	2.19	2.58	1.34	0.63	1.94	0.42	2.23
*mokI*	1.00	2.49	7.82	2.49	1.62	1.94	2.61	21.81

* Expression was determined by qRT-PCR relative to *glyceraldehyde 3-phosphate dehydrogenase* (*GAPDH*). Expression levels in M1 at 2 days served as the control group.

The expression of nine genes encoding different enzymes involved in monacolin K biosynthesis were verified by qRT-PCR (Fig 9). The expression patterns of the nine genes differed between M1 and M1-36. Moreover, *mokC*, *mokD*, *mokE*, *mokF*, *mokH* and *mokI* levels showed strong positive correlations with monacolin K concentration as compared to the observed expression profiles and previously measured monacolin K contents. These results suggest that differences in the expression patterns of *M*.*purpureus* genes account for monacolin K diversity.

**Fig 10:**
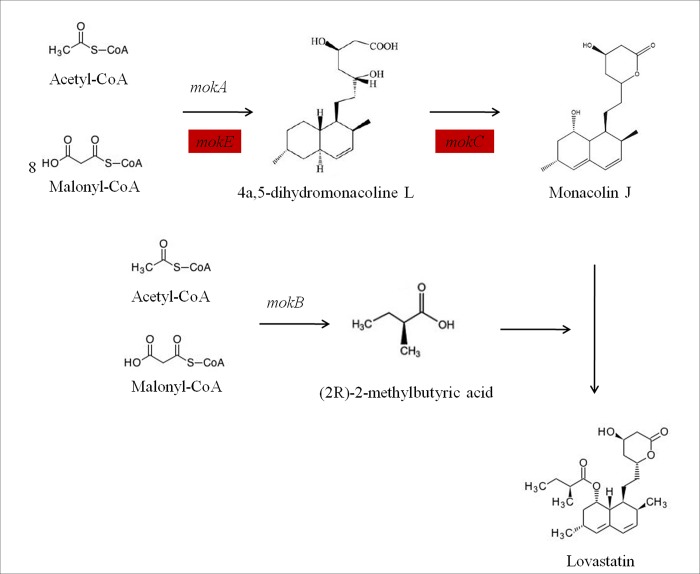
Monacolin K biosynthetic pathway in *M*. *purpureus*. The red rectangles indicate up-regulated genes including *mokE* and *mokC* genes.

To better understand the biological functions of genes on monacolin K biosynthesis in *M*. *purpureus*, monacolin K biosynthetic pathway were drawn (Fig 10). This result shows that *M*. *purpureus* M1-36 contained a higher monacolin K proportion than M1, suggesting that monacolin K synthesis was more active in M1-36 than in M1. The transcriptome analysis indicated that under high monacolin K production conditions, most genes in this polyketide synthase (PKS) cluster, especially the PKS gene (*mokD*), are expressed at high levels, which further supports the key role of this gene cluster in monacolin K synthesis. *MokD* and its homolog gene *ctnB* are belonged to the PKS gene clusters, and play as oxidoreductase in the synthesis pathway of their respective monacolin K and citrinin. Li et al found that the *ctnB*–deficient mutant in *M*. *aurantiacus* barely produced citrinin, indicating that *ctnB* gene is directly involved in citrinin biosynthesis [53]. Therefore, it is speculated that the *mokD* gene plays an important role in monacolin K synthesis. Currently, the function of *mokD* gene is being validated at our lab.

## Conclusions

In this study, we obtained the transcriptome information of *M*. *purpureus*, including functional annotation and classification, which could provide insight into the role of intracellular metabolic pathways [54]. In this study, we examined the functions of *M*. *purpureus* unigenes by BLAST searches against various databases [55]. Illumina HiSeq 2000 sequencing generated 60 million high-quality short reads, which were assembled into 29,713 assembled unigenes in *M*. *purpureus*. In summary, 122,599 unigenes showed a match with the GO annotation. Meanwhile, biosynthetic pathways involving some of these unigenes have been identified. These results contribute to existing sequence resources of *M*. *purpureus* and will be useful for studies on the biosynthesis of secondary metabolites such as aminoacyl-tRNA, nitrogen metabolism, and terpenoids in *M*. *purpureus*.

Owing to the economic and medicinal importance of *M*. *purpureus* [56], most studies on this organism have focused on the optimization of fermentation conditions, the physiological activity of secondary metabolites, and the PKS clusters genes of monacolin K biosynthesis. However, few *M*. *purpureus* genes have been characterized. One study found that *mokF* modulates monacolin K biogenesis in *M*. *purpureus*. In this study, nine genes (*mokA*-*mokI*) were found to be associated with the biosynthesis of monacolin K. The research of these genes could give us a deeper understanding on monacolin K biosynthesis and other secondary metabolic processes in *M*. *purpureus*.

## Supporting Information

S1 File *Monascus* transcriptomics data. (RAR)
